# Automated vertical cup-to-disc ratio determination from fundus images for glaucoma detection

**DOI:** 10.1038/s41598-024-55056-y

**Published:** 2024-02-24

**Authors:** Xiaoyi Raymond Gao, Fengze Wu, Phillip T. Yuhas, Rafiul Karim Rasel, Marion Chiariglione

**Affiliations:** 1 Department of Ophthalmology and Visual Sciences, The Ohio State University, Columbus, OH 43210 USA; 2 Department of Biomedical Informatics, The Ohio State University, Columbus, OH 43210 USA; 3 Division of Human Genetics, The Ohio State University, Columbus, OH 43210 USA; 4 College of Optometry, The Ohio State University, Columbus, OH USA

**Keywords:** Glaucoma, Vertical cup-to-disc ratio, Deep learning, YOLOv7, REFUGE dataset, Optic nerve diseases, Computational models

## Abstract

Glaucoma is the leading cause of irreversible blindness worldwide. Often asymptomatic for years, this disease can progress significantly before patients become aware of the loss of visual function. Critical examination of the optic nerve through ophthalmoscopy or using fundus images is a crucial component of glaucoma detection before the onset of vision loss. The vertical cup-to-disc ratio (VCDR) is a key structural indicator for glaucoma, as thinning of the superior and inferior neuroretinal rim is a hallmark of the disease. However, manual assessment of fundus images is both time-consuming and subject to variability based on clinician expertise and interpretation. In this study, we develop a robust and accurate automated system employing deep learning (DL) techniques, specifically the YOLOv7 architecture, for the detection of optic disc and optic cup in fundus images and the subsequent calculation of VCDR. We also address the often-overlooked issue of adapting a DL model, initially trained on a specific population (e.g., European), for VCDR estimation in a different population. Our model was initially trained on ten publicly available datasets and subsequently fine-tuned on the REFUGE dataset, which comprises images collected from Chinese patients. The DL-derived VCDR displayed exceptional accuracy, achieving a Pearson correlation coefficient of 0.91 (*P* = 4.12 × 10^–412^) and a mean absolute error (MAE) of 0.0347 when compared to assessments by human experts. Our models also surpassed existing approaches on the REFUGE dataset, demonstrating higher Dice similarity coefficients and lower MAEs. Moreover, we developed an optimization approach capable of calibrating DL results for new populations. Our novel approaches for detecting optic discs and optic cups and calculating VCDR, offers clinicians a promising tool that significantly reduces manual workload in image assessment while improving both speed and accuracy. Most importantly, this automated method effectively differentiates between glaucoma and non-glaucoma cases, making it a valuable asset for glaucoma detection.

## Introduction

Glaucoma is a chronic, progressive optic neuropathy that is the leading cause of irreversible blindness on a global scale^1–4^. It affects an estimated 70 to 90 million individuals worldwide and is responsible for approximately 4.5 million cases of blindness^5,6^. In the United States alone, the economic burden of caring for and treating glaucoma is staggering, amounting to nearly $2.86 billion annually. One of the most dangerous aspects of glaucoma is that it is asymptomatic in its early stages. A substantial proportion—nearly half—of those with the disease are unaware of their condition, even in developed countries. This lack of awareness may result in advanced disease with significant loss of vison at the time of initial diagnosis. Thus, early detection and intervention are paramount for mitigating vision loss due to glaucoma.

**Figure 1:**
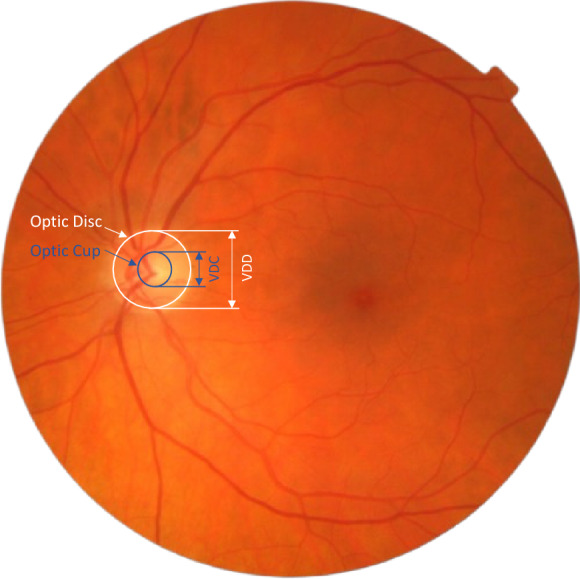
An example fundus image with marked optic disc and cup along with their corresponding vertical diameters. The optic disc is marked in white. The optic cup is marked in blue. VDD and VDC represent the vertical diameter of the optic disc and the vertical diameter of the optic cup, respectively.

The vertical cup-to-disc ratio (VCDR) is a critical structural indicator of the disease given the loss of superior and inferior neuroretinal rim thickness in glaucoma. VCDR is defined as the ratio between the vertical diameter of the optic cup and that of the optic disc within the optic nerve head. Figure 1 presents an example of a fundus image with marked optic disc and cup, along with their corresponding vertical diameters. Any elongation of the VCDR can elicit suspicion for glaucoma, and a VCDR value exceeding 0.7 may be an indicator for increased glaucoma risk^7^. Although eye care specialists often subjectively assess VCDR with clinical examination of the optic nerve or with fundus images, these manual techniques are both labor-intensive and subject to the variability of individual expertise with inter-grader correlations being only about 0.75^8–10^.

Past research has introduced various methodologies for automating measurement of the VCDR, ranging from manually crafted methods to more sophisticated deep learning (DL) algorithms. Examples of early methods include histogram matching^11^, fuzzy convergence and the Hough transform^12^, superpixel classification^13^, inpainting and active contour mode^14^, and *K*-means clustering and Gabor wavelet transform^15^, among others. In recent years, DL techniques, such as VGG^16^, ResNet^17^, DenseNet^18^, U-Net^19^, M-Net^20^, and Mask R-CNN^21^, have shown increasing promise in terms of estimation accuracy across various applications involving assessment of the optic disc, the optic cup, and the VCDR^22–28^. However, the rapid evolution of DL architectures opens new avenues for even more accurate VCDR estimations.

In light of these developments, our study introduces an automatic algorithm based on the cutting-edge YOLOv7^29^ DL model for detecting both the optic disc and the optic cup. Our results not only surpass existing state-of-the-art methods in terms of optic disc and cup detection but also provide superior VCDR estimations. By doing so, our algorithm has the potential to reduce the manual workload on clinicians while delivering a more objective and quantifiable VCDR assessment. Additionally, we address the often-overlooked issue of adapting a deep learning model trained on a specific population (e.g., European samples) for use on a different population (e.g., Chinese patients) for VCDR estimation. Most importantly, our DL-based VCDR values have also proven to be effective in distinguishing between glaucoma and non-glaucoma cases when applied to fundus images, thereby offering a promising tool for glaucoma detection.

## Results

**Figure 2:**
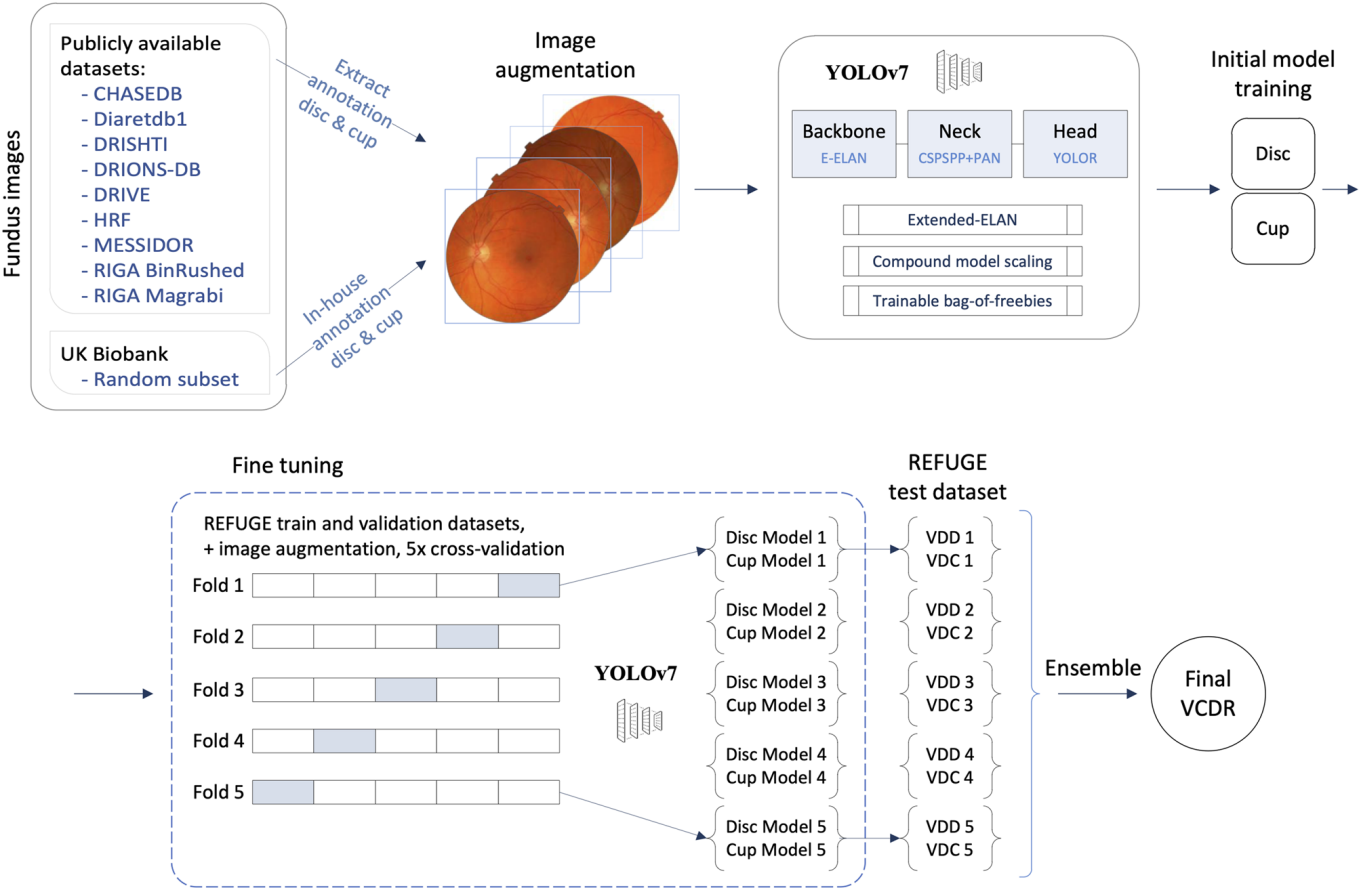
Flow chart of the study design. The flowchart outlines the systematic procedure for detecting the optic disc and optic cup, followed by the computation of the vertical cup-to-disc ratio (VCDR) using deep learning techniques. The entire pipeline consists of multiple stages—data collection, annotation, image augmentation, initial model training, fine-tuning, and ensemble techniques for final VCDR calculation. The top panel shows the initial model training, and the bottom panel shows the fine-tuning. The state-of-the-art YOLOv7 object detection architecture is used for detecting the optic disc and optic cup. Abbreviations: E-ELAN, extended efficient layer aggregation network; VDC, vertical diameter of the cup; VDD, vertical diameter of the disc; VCDR, vertical cup-to-disc ratio.

Figure 2 shows a flowchart of our study design, detailing a systematic procedure for optic disc and cup detection and VCDR computation using DL techniques. The entire pipeline consists of multiple stages, including data collection, annotation, image augmentation, initial model training, fine-tuning, and ensemble techniques for final VCDR calculation. The process begins with the collection of 10 publicly available datasets, predominantly originating from European countries. Following the annotation phase, image augmentation techniques are applied to enhance the diversity and scale of the datasets. The augmented images are fed into a YOLOv7 architecture to construct two separate models: one dedicated to the optic disc and the other to the optic cup. Post initial model training, fine-tuning is employed to refine the models to the REFUGE dataset, which consists of images collected from Chinese patients. This fine-tuning is carried out using a five-fold cross-validation approach. The outcome of the five-fold cross-validation is five distinct sets of models. Each set comprises a model for detecting the optic disc and another for detecting the optic cup. These models are then utilized to extract the vertical diameters of the optic disc and optic cup from the datasets. Finally, ensemble techniques amalgamate the outcomes from the five sets of models to derive a unified and robust VCDR.

Using our disc and cup models trained on the publicly available datasets, we tested their performance on the REFUGE test dataset (encompassing 400 images). We were able to accurately detect 100% of discs and cups in each image of the dataset. The Pearson correlation coefficient value between the derived and ground-truth VCDRs was 0.85 (95% CI 0.83–0.89, *P* = 4.10 × 10^–236^), demonstrating better degrees of agreement than human inter-grader correlations, which are only moderate with correlations around 0.75 based on previous reports^8–10^.

**Figure 3:**
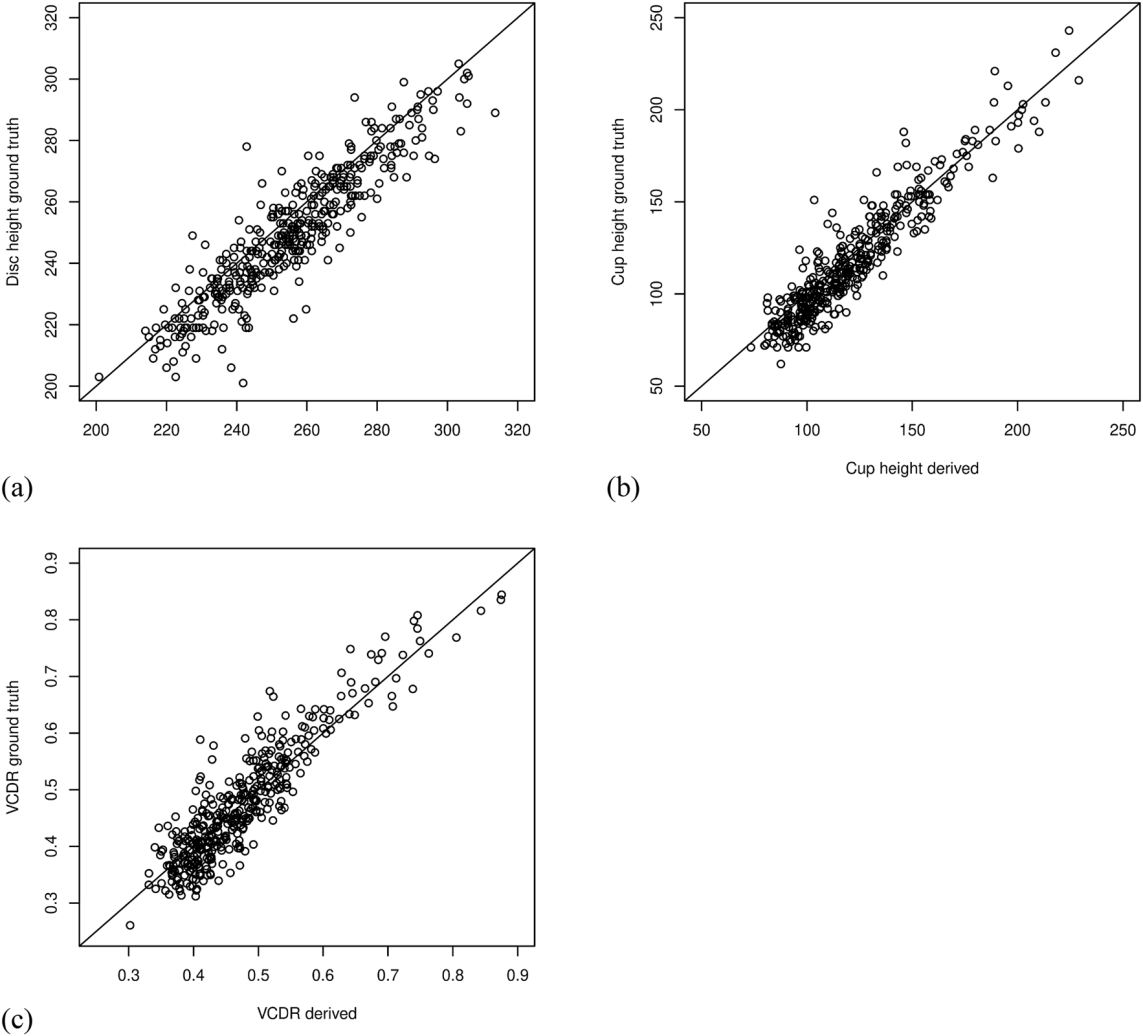
Pairwise plot comparing the derived vertical diameters of the disc and of the cup and derived VCDR with their corresponding ground truth values. X-axis denotes the deep learning derived value. Y-axis denotes the ground truth value. The diagonal line indicates a perfect match between the deep learning derived value and ground truth. (**a**) optic disc; (**b**) optic cup; (**c**) vertical cup-to-disc ratio (VCDR). Model performance was evaluated on the REFUGE test dataset.

To adapt our pre-trained models on the REFUGE data, we fine-tuned our DL models to better conform to the REFUGE dataset through fine-tuning. Figure 3 illustrates the derived and ground truth values pertinent to disc and cup detection, where Fig. 3a and b present the pairwise plots of the disc and cup heights versus their ground truth values, respectively. Meanwhile, Fig. 3c displays the pairwise plot of the derived versus ground-truth VCDR values. The plots show high correlations between our DL-derived values and growth truth. In particular, there is a solid correlation between the derived and ground-truth VCDRs of 0.91 (95% CI 0.89–0.92, *P* = 4.12 × 10^–412^), accompanied by a low MAE of 0.0347. A Bland–Altman plot further corroborates the close agreement between the derived and ground-truth VCDR values, as illustrated in Supplementary Fig. 1. Furthermore, our model scored DSCs of 0.9645 and 0.8937 for disc and cup segmentation, respectively. The high DSCs and the low MAE outperformed previous reports on the REFUGE dataset^30^ (Supplementary Table 1), including our previous report using Mask R-CNN^27^.

**Figure 4:**
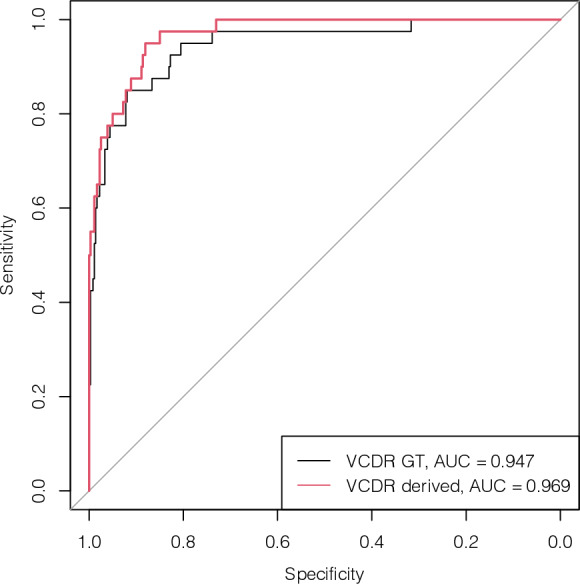
Receiver operating characteristic curves for predicting glaucoma. AUC curves for the prediction accuracy of two models: (1) deep learning-derived VCDR; (2) ground truth VCDR. Model performance was evaluated on the REFUGE test dataset. Abbreviations: AUC, area under the receiver operating characteristic curve; GT, ground truth; VCDR, vertical cup-to-disc ratio.

The effectiveness and robustness of our technique is further emphasized in Fig. 4, which presents glaucoma classification results obtained via logistic regression on VCDR. This figure displays the receiver operating characteristic (ROC) curve, with the x-axis denoting specificity and the y-axis denoting sensitivity. The closer the ROC curve is to the top-left corner, the better the model’s performance in glaucoma classification. Achieving an AUC of 0.969 (95% CI 0.95–0.99) from the derived VCDR, our model significantly outperformed ground-truth VCDR, which yielded a lower AUC of 0.947.

**Figure 5:**
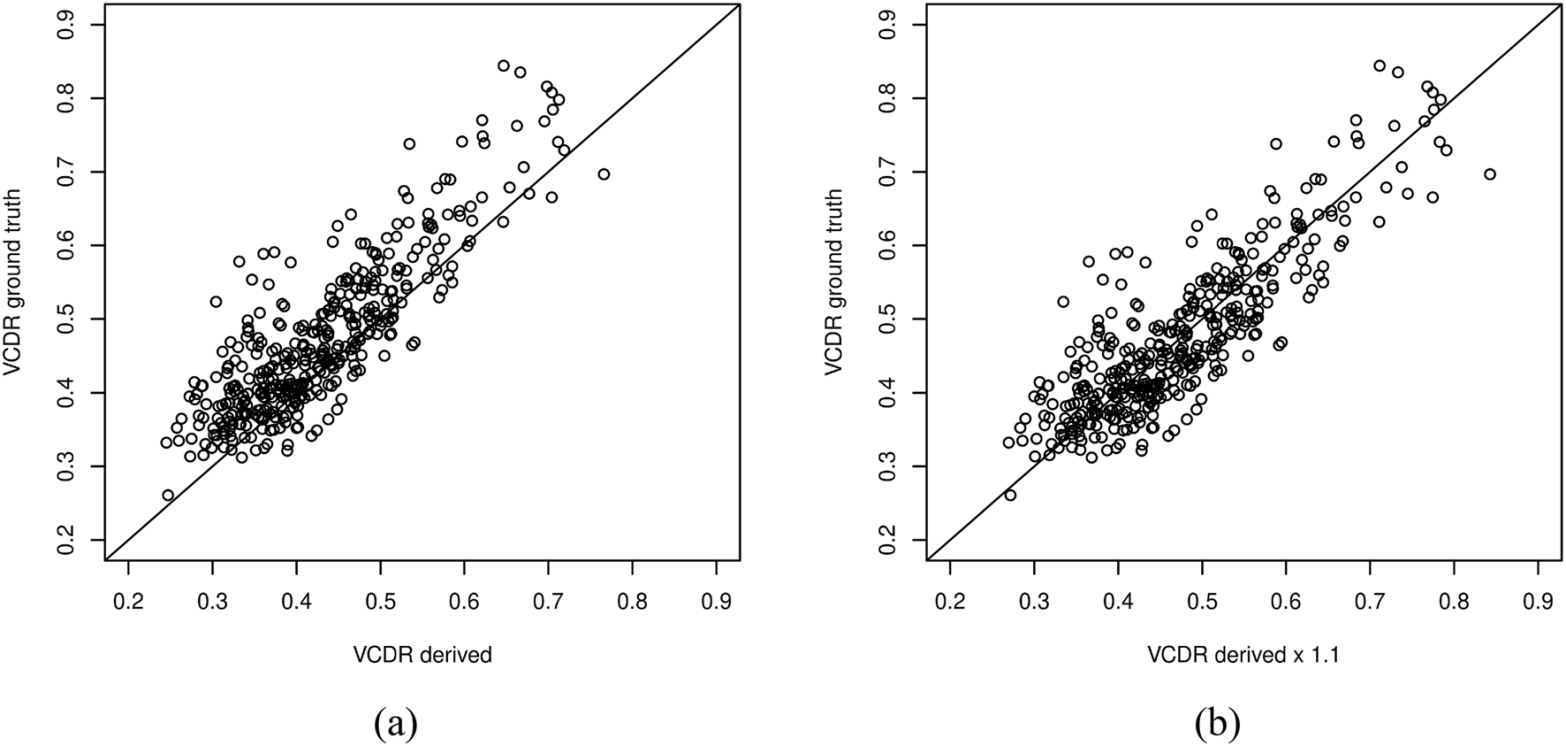
Pairwise plot comparing the directly derived and optimization calibrated VCDR versus the ground truth VCDR. The x-axis denotes (**a**) the directly derived VCDR (obtained from our initial model primarily trained on European samples) and (**b**) the optimization-calibrated VCDR (derived VCDR multiplied by the adjustment factor, 1.1 in this case). The y-axis denotes the ground truth VCDR. Model performance was evaluated on the REFUGE test dataset. Abbreviation: VCDR, vertical cup-to-disc ratio.

In addition to the fine-tuning approach, we also explored an optimization approach to calibrate the pre-trained DL-derived VCDR directly without relying on any additional DL-based techniques. For instance, Fig. 5a shows a pairwise plot comparing derived VCDR values with ground-truth VCDR values. These derived VCDR values are obtained from the initial model, which was primarily trained on the European samples. The results suggest a data shift (slightly above the diagonal line) that could potentially be corrected by an adjustment factor. To identify the optimal adjustment factor, we employed the optimization function, optim(), from R on the REFUGE validation dataset, consisting of 400 images independent of the REFUGE test dataset. We found the optimal adjustment factor to be 1.0947. As illustrated in Fig. 5b, this approach significantly improved alignment in the pair-wise plot between the adjusted VCDR (derived VCDR multiplied by 1.1) and the ground-truth VCDR. This procedure yielded an MAE of 0.0433, an accurate outcome (low MAE) achieved without the need for further training data or fine-tuning model training. This optimization strategy can be particularly valuable when the local DL or ground-truth resources for the target population are limited.

## Discussion

In the present study, we proposed a systematic procedure for optic disc and optic cup detection in fundus images using the state-of-the-art YOLOv7 DL architecture, followed by the computation of VCDR using DL techniques. Our pipeline consists of multiple stages—data collection, annotation, image augmentation, initial model training, fine-tuning, and ensemble techniques for final VCDR calculation. We trained our initial disc and cup detection models using publicly available dataset and subsequently refined them to a new population through fine-tuning. Our DL-derived VCDR results demonstrated a high degree of accuracy compared to the ground truth assessments by human experts. Furthermore, we developed an optimization approach capable of calibrating DL results for new populations, providing an alternative approach to fine-tuning in VCDR determination.

Our DL-derived VCDR gave highly accurate results when compared with human experts, achieving a correlation of 0.91 and an MAE of 0.0347. Earlier studies reported that the inter-grader correlations for VCDR estimates between human graders were only about 0.75^8–10^. The DL-derived VCDR can serve as a promising tool to assist clinicians in assessing the structure of optic disc and optic cup and VCDR estimation, saving clinicians from the time-consuming step of manual estimation. Furthermore, once the DL models are trained, the time for analyzing an individual image is minimal and the steps are fully automated. Hence, the DL models surpass human observers in this task in both accuracy and speed.

Addressing the applicability of pre-trained models, particularly those trained on European datasets, to diverse populations is crucial. In this study, we explored two approaches. First, we employed fine-tuning to adapt our pre-trained model to the REFUGE train and validation images, which were subsequently applied to the REFUGE test dataset. Second, we took an optimization approach, which used the results from our pre-trained model on the REFUGE images directly, and obtained highly accurate results, though slightly less so than the results from fine-tuning. This optimization approach is especially useful when computing resources and well-annotated datasets are limited.

In the context of VCDR estimation, which fundamentally involves determining the heights of the optic disc and cup, the task aligns naturally with object detection capabilities. This rationale led us to consider the YOLO (You Only Look Once) architecture as a suitable candidate. We found that YOLOv7 delivered highly accurate performance on real-world datasets encompassing diverse populations. Its efficacy and efficiency in our specific application ultimately guided our decision to utilize YOLOv7 for this study. The necessity for fine-tuning arose from the fact that the model's initial training data and the target images were sourced from different populations and captured using various camera types. Several factors contribute to the need for this retraining. These include, but are not limited to, variations in the populations from which the images were sourced (e.g., European versus Chinese populations), differences in camera equipment (such as Canon versus TOPCON), and disparities in image resolution. These variations can significantly affect the model's performance, making fine-tuning a crucial step to ensure higher detection accuracy in the target population.

Our study is not without limitations. Although we predominantly utilized YOLOv7 as the DL architecture. We also tested YOLOv5, ResNet^17^, and Mask R-CNN^21^ and found them to be less effective in real-world datasets from diverse populations in our tests. Many other DL architectures, such asYOLOv3^31^, U-Net^32^, M-Net^20^, and DenseNet^33^, can also be employed. An ensemble of results from these different DL architectures is likely to give better results. A comprehensive comparison of various architectures, including but not limited to YOLOv3, ResNet, and DenseNet, for automated VCDR estimation is beyond the scope of this research. For readers interested in such comparisons, we recommend the study by Park et al., 2020^34^. Our primary goal was to identify an effective method for automatic VCDR derivation from fundus images applicable to real-world datasets from diverse populations. In this regard, our approach using YOLOv7 proved to be highly effective. In some cases, the appearance of the optic disc may be oval or triangular in shape. For example, myopic eyes often present with tilted discs with substantial temporal sloping and peripapillary atrophy. In such instances, identifying the optic cup can be extremely challenging, even for human experts. While the REFUGE dataset includes myopic eyes, additional labels for co-existing morbidities were lost during the anonymization proces^30^. Consequently, our algorithm's performance on anomalous optic discs remains untested and warrants further investigation. Therefore, we note that our results are specific to the shapes of the optic discs present in the REFUGE dataset only. In exploring the AUC of glaucoma detection, we used VCDR only since that is the only optic nerve parameter that we assessed in this study. There are other parameters for glaucoma, such as retina nerve fiber layer defect, disc hemorrhage, vessel bayonetting, and lamina dot sign. The performance to classify glaucoma is currently limited to the REFUGE dataset and may vary when applied to other datasets. Further including image-based classification results is certain to increase the detection of glaucoma. Nevertheless, our study demonstrated the effectiveness, robustness, and its state-of-the-art performance of YOLOv7 in VCDR determination.

In summary, our novel approach for detecting one of the key structural features of the optic nerve head, namely the VCDR, in fundus images proved highly accurate when compared to human expert assessments. The proposed system could serve as an automated tool to derive VCDR from fundus images, alleviating the time-consuming labor of manual estimation. Our algorithm substantially eases the manual workload on eye specialists while furnishing a more objective and quantifiable VCDR assessment. Most importantly, our DL-based VCDR values have proven to be effective discriminators between glaucoma and non-glaucoma cases when applied to fundus images, thereby offering a promising tool for glaucoma detection.

## Methods

### Datasets

In this study, we utilized a composite of 10 publicly available datasets, amounting to a total of 2,402 color fundus images, to train our initial DL models for optic disc and cup detection. Table 1 provides an overview of each dataset, including specifics such as image count, resolution, field of view (FOV), capturing device, and data collection locales. The datasets range in size from 28 to 1,200 images, with MESSIDOR being the largest. Image resolutions span from as low as 565 × 584 to as high as 2745 × 1936 pixels. The FOV angles primarily fall into three categories: 30, 45, and 50 degrees. Various capturing devices, from handheld to analog to digital cameras from brands like Nidek, Canon, Topcon, have been used. The majority of the images were captured using non-mydriatic cameras, with most originating from European countries, except for around one hundred from India and nearly three hundred from Saudi Arabia.

**Table 1:** List of public datasets used for training our initial deep learning models.

Dataset	No. of images	Image resolution (pixels)	FOV	Camera	Data collection site
CHASEDB^37^	28	999 × 960	30°	Handheld fundus camera Nidek NM-200-D	England
DiaRetDB1^44^	89	1500 × 1150	50°	Digital fundus camera	Finland
Drishti-GS^45^	101	2049 × 1751	30°	UNS, dilated	India
DRIONS-DB^39^	110	600 × 400	30°	Color analogical fundus camera	Spain
DRIVE^38^	40	565 × 584	45°	Canon CR5 non-mydriatic 3CCD camera	The Netherlands
HRF^46^	45	3504 × 2336	45°	Canon CR-1 fundus camera	Czech Republic and Germany
MESSIDOR^47^	1200	2240 × 1488	45°	Topcon TRC NW6 non-mydriatic 3CCD camera	France
RIGA BinRushed^36^	195	2739 × 1584	45°	Canon CR2 non-mydriatic digital retinal camera	Saudi Arabia
RIGA Magrabi^36^	95	2745 × 1936	UNS	Topcon TRC 50DX mydriatic retinal camera	Saudi Arabia
UK Biobank subset^48^	500	2049 × 1536	45°	Topcon 3DOCT-1000 Mk 2 non-mydriatic fundus camera	United Kingdom

FOV: field of view, UNS: unspecified.

These datasets were initially collected for various research objectives. For example, MESSIDOR^35^ and DiaRetDB1^35^ aimed at diagnosing diabetic retinopathy, RIGA^36^ focused on glaucoma analysis, CHASEDB^37^, DRIVE^38^, and HRF^39^ were intended for retinal vessel segmentation. Additionally, Drishti-GS^40^ and DRIONS-DB targeted optic nerve head segmentation. Previous studies have already annotated these datasets for the optic disc and optic cup^36,41^, annotations that were utilized in this study.

Additionally, we employed the UK Biobank (UKB) dataset for model training. Detailed cohort information has been previously described^42,43^. In brief, UKB is a large-scale, ongoing population-based study involving adults aged 40–70 in the United Kingdom. About 95% of the UKB participants are of European ancestry. Color fundus images were taken from about 67,000 participants during the baseline data collection. We randomly selected 500 fundus images from this cohort for in-house annotation using LabelImg v1.4.0 and included them in our model training. Our access to this data was duly approved under application 23,424 and only fully de-identified data was utilized. Informed consent was obtained from the participants for their participation in the study by the UKB Committee upon recruitment. The study protocol was approved by The North West Multi-centre Research Ethics Committee. All methods present in this paper were performed in accordance with the Declaration of Helsinki.

For model evaluation, we used the Retinal Fundus Glaucoma Challenge (REFUGE) dataset^30^. This dataset was selected due to its high-quality ground-truth annotations from seven ophthalmologists, as well as its glaucoma labels, which are based on a comprehensive evaluation of clinical records. This dataset comprises 1,200 color fundus images from Chinese patients, divided into 400 train, 400 validation, and 400 test images, each captured with specific cameras and resolutions. The train subset was captured using Zeiss Visucam 500 and have an image resolution of 2124 × 2056 pixels. The validation and test subsets were captured using Cannon CR-2 and have an image resolution of 1634 × 1634 pixels. The REFUGE dataset enabled us to validate our DL models on a distinct population with different capturing devices and resolutions.

### YOLOv7 object detection DL model

For optic disc and cup detection, we employed YOLOv7^29^, a state-of-the-art algorithm for real-time object detection. This seventh version of the YOLO (You Only Look Once) model boasts improved speed and accuracy. The architectural incorporates novel features like E-ELAN (extended efficient layer aggregation network) and compound model scaling to enhance learning ability. Additionally, trainable bag-of-freebies, such as planned re-parameterized convolution, coarse for auxiliary and fine for lead loss, further enhance the accuracy of object detection. We trained separate models for identifying the optic disc and cup from color fundus images using YOLOv7.

### Train YOLOv7 models for disc and cup detection

To train our models, we processed the fundus images to form square dimensions. This involved either adding black borders or cropping to transform the original rectangular fundus images into square images. This step of creating square images is a common requirement for most DL architectures, which typically necessitate uniform input dimensions. This normalization process did not artificially alter the geometry of the optic nerve head. The integrity of the anatomical structures within the images, including the optic nerve head, was maintained throughout this standardization procedure. We resized the ten publicly available datasets to a uniform dimension of 1536 × 1536 pixels and performed image augmentation such as horizontal flipping and brightness adjustments. The data was then partitioned into training and validation sets at an 80:20 ratio. Subsequently, we trained our models from scratch using the YOLOv7-x architecture, with varying batch sizes and epochs based on the specific task. For the training the disc detection model, we used a batch size of 12 and 150 epochs. We then extracted the disc region at the size of 384 × 384 and trained the cup detection model with a batch size of 128 and 300 epochs. The delineation of the 384 × 384 region for cup detection was defined automatically, based on the results of the disc detections. Upon detecting an optic disc, we obtained its central coordinates (x_center, y_center). These central coordinates, x_center and y_center, were then utilized as the center for the 384 × 384 region designated for cup detection. All training was conducted on NVIDIA Tesla V100 GPUs using Python v3.7.

### Fine-tuning

To adapt our above models for the REFUGE dataset, we employed a fine-tuning approach, training them on 800 images from the REFUGE training and validation sets and evaluating on its 400-image test set. We utilized five-fold cross-validation, applied image augmentations, and trained five sets of models for the optic disc and cup detections. Subsequently, these models were ensembled to derive the final VCDR. All the fine-tuning processes were conducted on NVIDIA Tesla V100 GPUs using Python v3.7 as well.

### Evaluation metrics

To compare DL-detected optic disc and cup with their ground truth, we applied the Dice similarity coefficient (DSC), which is defined as:

$$DSC=2\frac{{DL}_{k}\cap {GT}_{k}}{{DL}_{k}{\cup GT}_{k}}$$

where GT_k_ corresponds to ground truth and DL_k_ is the predicted optic disc/cup region, and k = disc or cup. The average of DSC was computed from all the REFUGE test dataset images.

For assessing the VCDR estimations, we used the mean absolute error (MAE), which is defined as:

$$MAE= \frac{1}{n}{\sum }_{i=1}^{n}\left|{VCDR}_{{DL}_{i}}-{VCDR}_{{GT}_{i}}\right|$$

where ${VCDR}_{{DL}_{i}}$ is the VCDR value calculated using predicted optic disc and cup and ${VCDR}_{{GT}_{i}}$ using ground truth masks, with VCDR being defined as: $VCDR=VDC/VDD$, where $VDC$ and $VDD$ are the vertical diameters of the optic cup and disc, respectively.

Additionally, we utilized logistic regression and the area under the receiver operating characteristic curve (AUC) metric for evaluating the efficacy of DL-derived VCDR values in glaucoma classification. The statistical analyses were performed using R (v3.6.3).

### Optimization

We also developed an optimization strategy as an alternative to fine-tuning. This method required minimal computational resources and was applied to adapt our initial models trained on a specific population (images primarily from European samples) for use on a different population, such as images from Chinese patients and captured using a different camera in this case. We used the REFUGE validation dataset (independent of the test dataset) to calibrate the VCDR values derived from the REFUGE test dataset. We use the optim() function from R to estimate an optimization factor with the evaluation criterion to minimize the MAE between the derived VCDR and ground-truth VCDR. Then, we applied this optimization factor to all the DL-derived VCDR values of the test dataset from the pre-trained models. In contrast to the much more computational and ground truth resources needed for fine-tuning deep learning, this approach can serve as an effective alternative option when DL or the ground truth resources are limited in the target population.

### Supplementary Information

Supplementary Information.

## Data Availability

The data used in this paper is publicly available except the UKB data which was obtained via contract using application ID #23,424. Applications to access the data can be completed at: https://www.ukbiobank.ac.uk/enable-your-research/apply-for-access. Informed consent was obtained from the participants for their participation in the study by the UKB Committee upon recruitment. The study protocol was approved by The North West Multi-centre Research Ethics Committee. *Web Resources:* The URLs for downloaded data and programs: CHASE_DB, https://blogs.kingston.ac.uk/retinal/chasedb1/, DiaRetDB1, https://www.kaggle.com/datasets/nguyenhung1903/diaretdb1-v21, DRIONS-DB, http://www.ia.uned.es/~ejcarmona/DRIONS-DB.html, Drishti-GS, http://cvit.iiit.ac.in/projects/mip/drishti-gs/mip-dataset2/Home.php, DRIVE, https://drive.grand-challenge.org/, HRF, https://www5.cs.fau.de/research/data/fundus-images/, LabelImg, https://pypi.org/project/labelImg/1.4.0/, MESSIDOR, https://www.adcis.net/en/third-party/messidor/, REFUGE, https://refuge.grand-challenge.org/, RIGA, https://deepblue.lib.umich.edu/data/concern/data_sets/3b591905z, UK Biobank, https://www.ukbiobank.ac.uk, YOLOv7, https://github.com/WongKinYiu/yolov7.
