# Supplementary material for: Automated vertical cup-to-disc ratio determination from fundus images for glaucoma detection
Source: Sci Rep. 2024 Feb 24;14:4494. doi: 10.1038/s41598-024-55056-y (PMC10891153; doi:10.1038/s41598-024-55056-y)
Supplement: Supplementary file 1 — Supplementary Information. [file 41598_2024_55056_MOESM1_ESM.docx]

**Supplementary information**

**Supplementary Table 1. Results of the proposed method compared to REFUGE top ranked teams**

| **Method** | **Training set** | **Disc Avg.**  **DSC** | **Cup Avg.**  **DSC** | **VCDR**  **MAE** |
| --- | --- | --- | --- | --- |
| **Proposed** | REFUGE training and validation sets through transfer learning | 0.9645 | 0.8937 | 0.0347 |
| REFUGE rank #1 | REFUGE  training and validation sets | 0.9602 | 0.8826 | 0.0450 |
| REFUGE rank #2 | REFUGE  training set and ORIGA | 0.9464 | 0.8837 | 0.0414 |
| REFUGE rank #3 | REFUGE  training set | 0.9525 | 0.8728 | 0.0456 |

Note: The training datasets used varied among different studies. For a comparison utilizing only the REFUGE dataset, we recommend our previous study^1^, which employed the Mask R-CNN architecture and achieved superior metrics compared to the teams in the REFUGE challenge.

Abbreviations: Avg, average; DSC, dice similarity coefficient; MAE, mean absolute error; VCDR, vertical cup-to-disc ratio.

**Supplementary Figure 1. Bland-Altman agreement plot comparing derived and ground-truth VCDR values**


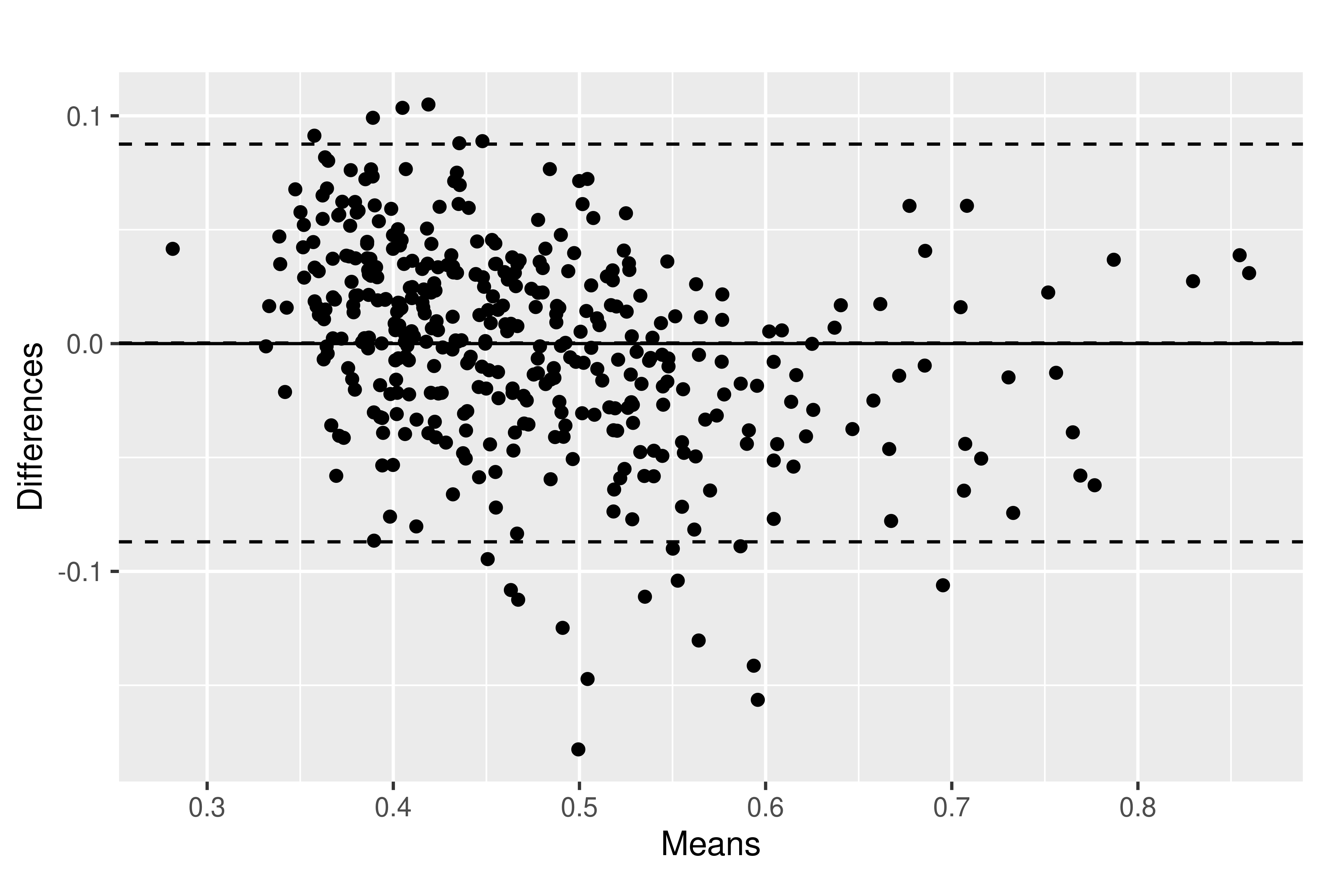


This figure shows the Bland-Altman plot illustrating the agreement between derived and ground-truth VCDR values. Dotted lines mark the limits of agreement, ranging from -1.96s to +1.96s, where 's' is the standard deviation of the differences between the two sets of VCDR values. Overall, the plot indicates the close agreement between the derived and ground-truth VCDR. The plots were generated using the R function blandr.draw().

Abbreviation: VCDR, vertical cup-to-disc ratio.

References

1 Wu, F., Chiariglione, M. & Gao, X. R. Automated Optic Disc and Cup Segmentation for Glaucoma Detection from Fundus Images Using the Detectron2's Mask R-CNN. *2022 International Symposium on Multidisciplinary Studies and Innovative Technologies (ISMSIT)*, 567-570, doi:10.1109/ISMSIT56059.2022.9932660 (2022).
